# Choledochal Cyst in the Context of Sickle Cell Disease: A Case Report

**DOI:** 10.7759/cureus.64568

**Published:** 2024-07-15

**Authors:** Pankaj Gharde, Pramita M Gharde, Meenakshi Yeola Pate, Yashwant Lamture, Harshal Tayade, Varun Kulkarni, Kavyanjali Reddy

**Affiliations:** 1 Surgery, Jawaharlal Nehru Medical College, Datta Meghe Institute of Higher Education and Research, Wardha, IND; 2 Community Medicine, Jawaharlal Nehru Medical College, Datta Meghe Institute of Higher Education and Research, Wardha, IND; 3 Surgery, All India Institute of Medical Sciences, Mangalagiri, Mangalagiri, IND

**Keywords:** endoscopy ercp, congenital abnormalities, obstructive jaundice, sickle cell disease, choledochal cyst

## Abstract

Choledochal cyst is a congenital pathology with an uncommon anomaly associated with common complaints of an abdominal lump and hepatic dysfunction. It may be presented equally in any phase of life, be it childhood, adolescence, or adulthood, and is majorly detected by ultrasonography (USG) on the appearance of primary symptoms in the hepato-biliary system. It has a classical triad consisting of a lump in the upper quadrant on the right side of the abdomen, pain in the upper part of the abdomen, and obstructive jaundice. A few of the clinical features overlap with sickle cell disease.

A 30-year-old male patient with sickle cell anemia was diagnosed eight years ago. The patient was diagnosed with a choledochal cyst with the clinical presentation of abdominal pain, nausea, and vomiting, which hampered his routine life. Due to symptomatic recurrence, the patient was subjected to USG (abdomen), which showed a dilated common bile duct (CBD) and dilated intrahepatic biliary radicals. This is a rare case presentation with both sickle cell disease and choledochal cyst, which are symptomatically similar. Based on history, risk factor analysis, and diagnostic findings, the patient was advised to have a Roux-en-Y hepatico-jejunostomy.

Endoscopic retrograde cholangiopancreatography (ERCP) and magnetic resonance cholangiopancreatography (MRCP) are the investigations of choice, with the better being MRCP. ERCP is a therapeutic and diagnostic modality that helps in the removal of CBD calculus and the placement of a stent. There may be increased bilirubin, showing features of obstructive jaundice in alcoholic stools. In surgical management, which is of total excision of the cyst, there are vital structures in proximity. The patients with these complaints need to be evaluated thoroughly, and detailed clinical examination and proper radiological investigations will be performed. Roux-en-Y hepatico-jejunostomy with cyst excision in toto is the procedure of choice.

## Introduction

Choledochal cyst is one of the uncommon congenital abnormalities of the hepatico-biliary tree. It involves the hepatobiliary tree, which may be intrahepatic and extra-hepatic. A congenital condition presenting at birth or during early childhood, less than 25% present in adulthood. It presents primarily in females and younger children, comparatively more frequently in Eastern Asia. Although benign, it may present with severe complications including gallbladder stones, cholangitis, choledocholithiasis, and pancreatitis with an increased risk of malignant changes. The incidence of malignancy is from 2.5% to 21%, and the risk of malignancy is higher in the Eastern population while it is reported to be higher in the Western world [1]. The presentation is not common and specific; it has a classical triad of features consisting of vague pain in the abdomen, a lump in the abdomen in the upper quadrant of the right side, and an obstructive type of jaundice encountered in younger children. Many clinical features overlap with those of sickle cell disease [2,3]. Sickle cell disease may result in vaso-occlusive events leading to end-organ damage to the kidneys and liver, necrosis of the femoral head, and splenomegaly. Hemolysis can result in pain in the abdomen, jaundice, and cholelithiasis [3].

Investigations are not very specific for the absolute diagnosis of a choledochal cyst. In the prenatal period, a choledochal cyst can be identified as a cystic structure at the porta hepatis on abdominal sonography, which gives good information regarding the type of cyst, its location, and echotexture of the liver, and the cause cannot be identified in 35% of patients. Computed tomography (CT) scan has a sensitivity of around 90%; it can also detect intrahepatic dilatation; CT cholangiography helps plan surgical approaches. Technetium-99 hepatobiliary iminodiacetic acid (HIDA) scan has a sensitivity of 100% in the case of intrahepatic ductal dilatation. Then comes magnetic resonance cholangiopancreatography (MRCP); it is a gold standard investigation with a sensitivity of nearly 100%; it is safe, non-ionizing, with no bleeding, no perforation, no cholangitis, or examination-induced pancreatitis, which otherwise may occur in endoscopic retrograde cholangiopancreatography (ERCP). ERCP is the ultimate diagnostic tool; the only disadvantage is that it exposes the patient to ionizing radiation; it is both diagnostic and therapeutic; one more advantage is that endoscopic sphincterotomy of the duct is possible [4]. The management is cyst excision and Roux-en-Y hepatico-jejunostomy. Complications of a choledochal cyst can be synchronous with acute pancreatitis, portal hypertension, choledocholithiasis, cholangitis, and jaundice. All these parameters are controlled, and the surgery is planned to get desirable results [5].

## Case presentation

**Figure 1:**
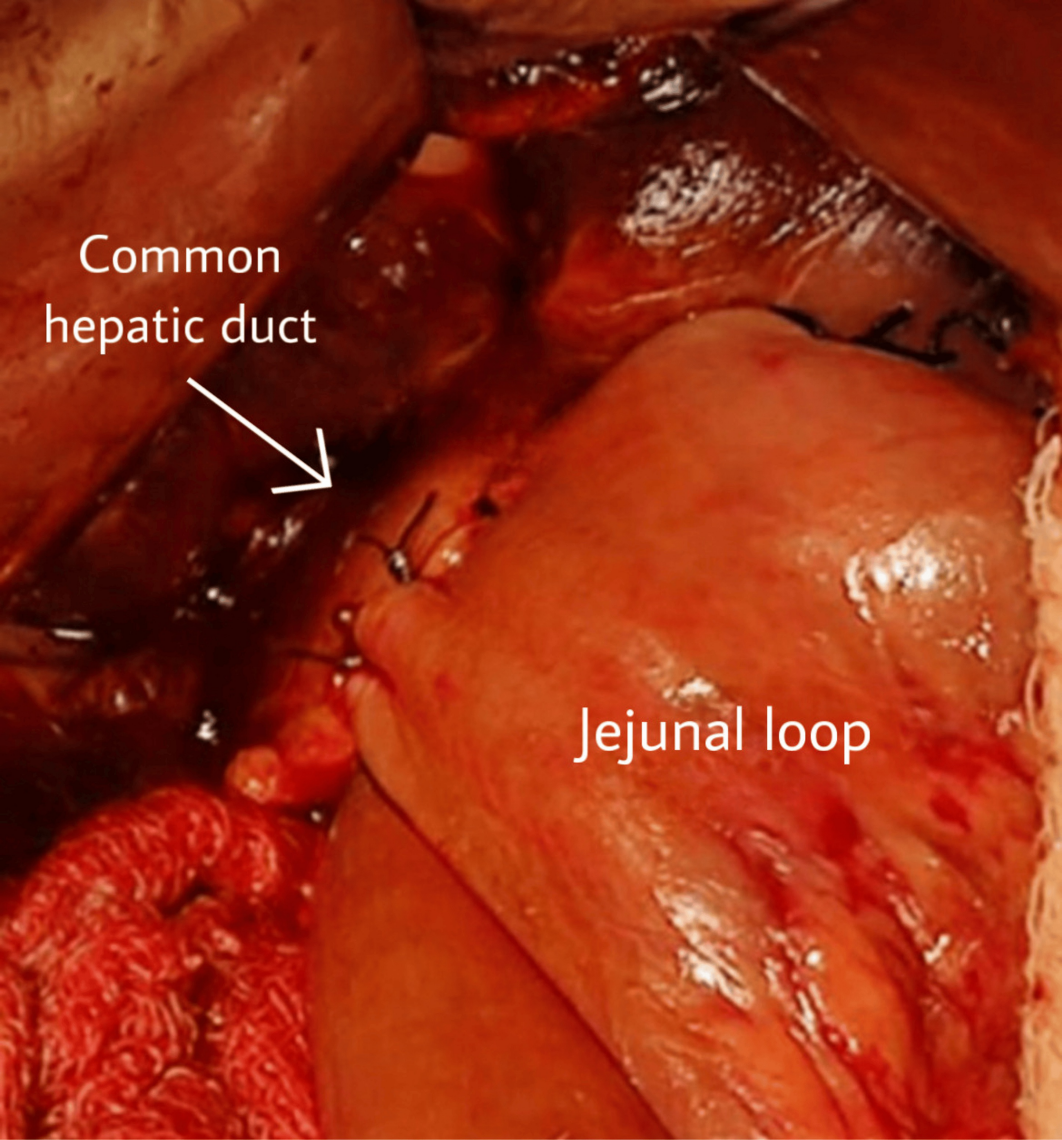
Anastomosis between the jejunum and common hepatic duct

A 30-year-old patient from central India came to us in the Outpatient Department and presented with pain in the right upper abdomen with sudden onset and gradually increasing in intensity; it radiated to the back, aggravated on walking, and was relieved on rest. The patient has had jaundice for the last two months with no history of fever. The vitals of the patient were normal. He was first diagnosed in 2012, and since then, he has undergone multiple conservative management treatments with a history of multiple admissions and procedures. He was not operated on in his earlier consultations, as it was a high-risk case because he also had sickle cell anemia of the SS pattern. He was admitted for blood transfusions at different centers. The final diagnosis of the patient was choledocholithiasis with type IA choledochal cyst with sickle cell anemia SS pattern. He was diagnosed with sickle cell disease at the age of five years; in 2012, he was diagnosed with choledocholithiasis with a dilated common bile duct (CBD). He underwent ERCP with stenting, and later, in the same year, he went for open cholecystectomy with CBD exploration, and then the stent was removed. In 2017, the patient was again diagnosed with choledocholithiasis with a dilated CBD, and again, ERCP with stenting was done. In November 2017, he was investigated for recurrent abdominal pain and again showed a picture of choledocholithiasis with a dilated CBD, for which removal of the previous stent, balloon sweep, and joint bile duct clearance was done. In August 2020, the patient presented to us with similar complaints of jaundice and pain in the abdomen. At this time, ultrasonography (USG) showed CBD dilatation with a diameter of 12 mm, and intrahepatic biliary radicals were dilated. For further evaluation, MRCP was done, which revealed focal dilatation of the extra-hepatic duct and CBD throughout the course, with evidence of two large intraluminal filling defects of 16 x 15 x 13 mm and 18 x 18 x 15 mm in the proximal CBD with ERCP stent in situ, suggestive of choledocholithiasis with type 1A choledochal cyst. Alanine transferase and gamma-glutamyl transferase levels on biochemical evaluation were elevated with a raised serum bilirubin. The patient was anemic, and biomedical markers were awaited to reach the normal range before the operative procedure. The procedure was planned as there was a component of cholangitis, which was present after supportive drugs and antibiotics management. He underwent surgery after 15 days. After the procedure was explained to the patient and relatives, the patient was kept well hydrated; informed written consent was obtained, and the patient was shifted to the operating room. The CBD was found to be dilated on exploration, and the CBD and the hepatic duct were dilated. They were dissected and freed from the sides and separated just below the junction of the common hepatic duct and Roux-en-Y loop of the jejunum raised from the retro colic area and hepatico-jejunostomy (Figure 1).

**Figure 2:**
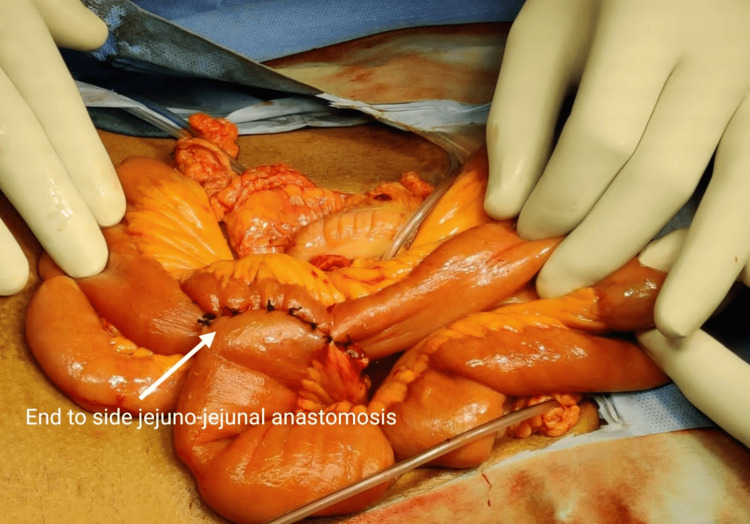
Anastomosis between the duodenum and common bile duct

**Figure 3:**
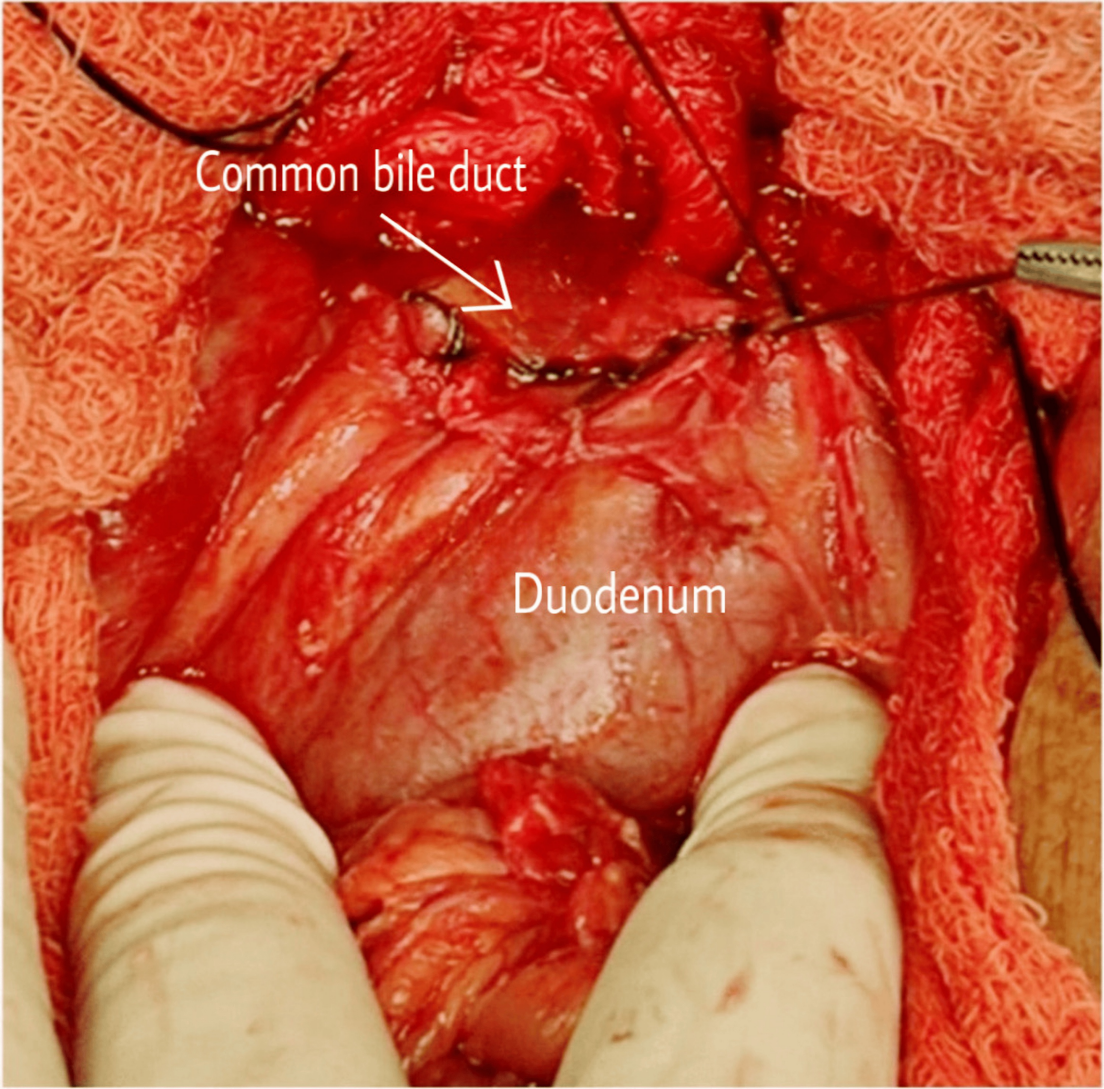
Anastomosis between the duodenum and common bile duct

A jejunostomy was done, and the duodenal end of the CBD was closed (Figures 2-3).

The patient was discharged after 10 days and is under monthly follow-ups for six months. The patient did not have any complaints regarding the procedure.

## Discussion

A choledochal cyst is a rare anomaly associated with common complaints of a lump in the abdomen and hepatic dysfunction. The main clinical features experienced by patients are nausea, vomiting, and abdominal pain. The case involves a mass in the upper quadrant on the right side. It is commonly observed in infants and children as they tend to play in groups. The characteristic findings of pain in the abdomen, a lump in the upper abdomen, and jaundice occur in 0-22% of cases. Many patients do not present with these findings, making it challenging to diagnose choledochal cysts. This patient is one of them because there was no lump in the abdomen, but jaundice was present with abdominal pain, which, on further evaluation, was found to be sickle cell disease with the SS pattern. Increased bilirubin may show features of obstructive jaundice, acholic stools, elevated serum alkaline phosphatase, gamma-glutamyl transferase, and prothrombin time, which may indicate a bleeding disorder [6].

Regarding imaging modalities, ultrasound is an excellent initial investigation for the intrahepatic and extrahepatic biliary systems; it can diagnose choledocholithiasis, cholangitis, cystic structures, and malignant changes. However, it cannot differentiate between malignant and non-malignant conditions. A CT scan can also be considered for a second opinion; it is less commonly required as the ducts are obscured by gases, and the cyst's biliary origin cannot be assured. The reason might be the presence of pseudocysts of the pancreas, omental cysts, mesenteric cysts, and hepatic cysts, which may be part of the differential diagnosis. A CT scan has a potential risk of ionizing radiation. ERCP and MRCP are now the investigations of choice, with MRCP being the preferred option. ERCP is a therapeutic and diagnostic modality that helps in the removal of CBD calculus and the placement of a stent. On the other hand, MRCP is the best diagnostic tool as it is non-ionizing, highly safe, and accurate, with sensitivity on par. The only disadvantage is its inability to detect ductal anomalies or minor choledochoceles. MRCP requires breath-holding, which can be done in adults but cannot be guaranteed in infants and small children. MRCP is preferred over ERCP when diagnosed by USG [7].

Todani's classification is a modified version of the famous Alonso-Lej criteria; it was introduced in 1977 [8,9]. According to it, type IA is a cystic dilatation of the extra-hepatic biliary tract, and the gall bladder opens into the main hepatic duct; type IB is focal segmental dilatation in the biliary tree in the extra-hepatic part, with the gall bladder opening into the biliary duct; type IC is regular fusiform dilatation of the duct from the pancreaticobiliary junction to the intrahepatic biliary tract and is proximal to the cyst; type II is a diverticulum originating from the extra-hepatic biliary tract, connected through a narrowed peduncle; type III is dilatation related to the duodenal wall in the distal part, with the outer wall possibly having duodenal mucosa, and the inner wall having duodenal or biliary epithelium; type IVA involves multiple intrahepatic and extra-hepatic dilatations of the structures, which can be fusiform, cystic, or irregular; type IVB involves multiple dilatations, including only the extra-hepatic biliary tract; type V, also known as Caroli's disease, has numerous intrahepatic cystic or saccular dilatations.

The risk of malignancy reported is 2.5-21% in Western countries, which might be overestimated compared to the Eastern or Asian population, where the risk is higher. The risk of malignancy increases with age; in the first decade of life, it is 0.7%, which increases to 10% in the second decade, and after 60 years, it can increase up to 50%. The reason behind the malignant change is the stasis of pancreatic secretions in the biliary tract, which causes inflammation, leading to dysplasia, metaplasia, and then cancer [1].

When it comes to surgery, it involves total excision of the cyst; there are vital structures in proximity. There are chances of adhesion to the pancreas and portal vein (which drains 75% of blood to the liver) due to recurrent attacks of cholangitis; in these circumstances, there are risks of bleeding and pancreatic leak. These cases are complex for complete excision. If an intrapancreatic choledochal cyst is not resected, it might result in dead space and anomalous pancreaticobiliary communication. The regurgitation of intestinal contents into the pancreatic duct may lead to inflammation, calculus formation, and an increased risk of malignant change. Total excision is the only solution to the choledochal cyst. Roux-en-Y hepatico-jejunostomy is one of the procedures. Cholangitis is common in patients in whom the cyst is not excised. The reflux of duodenal juice into the pancreatic duct activates inactivated pancreatic enzymes on the side of the pancreas, leading to pancreatitis and the progression of the choledochal cyst. Frequent episodes of regurgitation may result in stenosis of the pancreaticoduodenal and pancreaticobiliary junctions, causing frequent bouts of cholangitis and pancreatitis. The proximal part of the distal duct is excised and closed. Choledochoceles can be managed by trans-duodenal sphincterotomy, excision sphincteroplasty, or pancreatico-duodenectomy [10].

## Conclusions

It is a rare entity, yet common in the Asian population. There are chances of malignant change depending on its type; proper treatment prevents complications and results in favorable outcomes. Malignancy is also rare, but long-term follow-up is needed. Imaging and appropriate screening through ERCP (less preferred) and MRCP should be done to delineate the cyst, and if other anomalies are present, they should be handled appropriately. All biochemical markers should be controlled, and appropriate surgery should be planned depending on the type of cyst. Although there is no consensus on laparoscopic or open cholecystectomy, Roux-en-Y hepatico-jejunostomy with cyst excision in toto is the procedure of choice.
